# Predicting Symptoms of Depression and Anxiety Using Smartphone and Wearable Data

**DOI:** 10.3389/fpsyt.2021.625247

**Published:** 2021-01-28

**Authors:** Isaac Moshe, Yannik Terhorst, Kennedy Opoku Asare, Lasse Bosse Sander, Denzil Ferreira, Harald Baumeister, David C. Mohr, Laura Pulkki-Råback

**Affiliations:** ^1^Department of Psychology and Logopedics, Faculty of Medicine, University of Helsinki, Helsinki, Finland; ^2^Department of Research Methods, Ulm University, Ulm, Germany; ^3^Department of Clinical Psychology and Psychotherapy, Institute of Psychology and Education, Ulm University, Ulm, Germany; ^4^Center for Ubiquitous Computing, University of Oulu, Oulu, Finland; ^5^Department of Rehabilitation Psychology and Psychotherapy, Institute of Psychology, University of Freiburg, Freiburg, Germany; ^6^Department of Preventive Medicine, Center for Behavioral Intervention Technologies, Northwestern University, Chicago, IL, United States

**Keywords:** digital phenotyping, predicting symptoms, depression, anxiety, mobile sensing

## Abstract

**Background:** Depression and anxiety are leading causes of disability worldwide but often remain undetected and untreated. Smartphone and wearable devices may offer a unique source of data to detect moment by moment changes in risk factors associated with mental disorders that overcome many of the limitations of traditional screening methods.

**Objective:** The current study aimed to explore the extent to which data from smartphone and wearable devices could predict symptoms of depression and anxiety.

**Methods:** A total of *N* = 60 adults (ages 24–68) who owned an Apple iPhone and Oura Ring were recruited online over a 2-week period. At the beginning of the study, participants installed the *Delphi* data acquisition app on their smartphone. The app continuously monitored participants' location (using GPS) and smartphone usage behavior (total usage time and frequency of use). The Oura Ring provided measures related to activity (step count and metabolic equivalent for task), sleep (total sleep time, sleep onset latency, wake after sleep onset and time in bed) and heart rate variability (HRV). In addition, participants were prompted to report their daily mood (valence and arousal). Participants completed self-reported assessments of depression, anxiety and stress (DASS-21) at baseline, midpoint and the end of the study.

**Results:** Multilevel models demonstrated a significant negative association between the variability of locations visited and symptoms of depression (beta = −0.21, *p* = 0.037) and significant positive associations between total sleep time and depression (beta = 0.24, *p* = 0.023), time in bed and depression (beta = 0.26, *p* = 0.020), wake after sleep onset and anxiety (beta = 0.23, *p* = 0.035) and HRV and anxiety (beta = 0.26, *p* = 0.035). A combined model of smartphone and wearable features and self-reported mood provided the strongest prediction of depression.

**Conclusion:** The current findings demonstrate that wearable devices may provide valuable sources of data in predicting symptoms of depression and anxiety, most notably data related to common measures of sleep.

## Introduction

Depression and anxiety are leading causes of disability worldwide, with estimated lifetime prevalence rates of 20% (1). Whilst the majority of individuals with depression and anxiety are treated in primary care settings, over 50% of people are not recognized or adequately treated (2, 3). Given the adverse health outcomes and costs associated with untreated conditions and the recent increase in the prevalence of common mental disorders (4–6), adequate diagnosis and timely treatment of depression and anxiety has become an urgent priority.

Traditionally, researchers have relied on questionnaire data administered by a clinician or self-reported to assess an individual's mental health. However, these methods may be limited in their ability to detect the moment-by-moment changes in psychological factors that is required for preventative measures and rapid interventions. First, questionnaires often take place sporadically, with long intervals between them, during which time symptoms may change considerably. Second, these questionnaires often rely on retrospective evaluations and, as such, are prone to recall bias (7, 8). Third, there may be a tendency for respondents to provide socially-desirable answers (9, 10). Finally, patients typically only meet with a clinician or undertake assessments once the symptoms have already progressed to a certain level of severity, making prevention far more challenging.

Smartphone devices may offer a unique opportunity to overcome some of these limitations. Equipped with an array of sensors, smartphones unobtrusively provide a continuous stream of data related to an individual's mental health, including location, smartphone usage behavior, physical activity and social interactions (11, 12). This moment-by-moment quantification of the individual-level human phenotype *in situ* using data from personal digital devices is referred to as “digital phenotyping” (13, 14). There is now a growing body of research demonstrating that digital phenotyping data may enable the identification of people suffering from or at risk of developing mental disorders, in some cases even before symptoms are visible (or detectable) using traditional methods (11, 15–18).

One source of data that has yielded promising results in identifying those suffering from mental disorders is location data derived from smartphone global positioning systems (GPS). Saeb et al. (19), e.g., found that regularity of participants' 24-h movement patterns (*r* = −0.63), the variance of locations visited (*r* = −0.58) and the proportion of time spent at home (*r* = 0.49) were related to depressive symptom severity in a non-clinical population (19). Beiwinkel et al. (20) found that the total distance traveled had a significant negative relationship with clinical manic symptoms in patients diagnosed with bipolar disorder (beta = −0.37). Finally, in a meta-analytic review of studies assessing the correlation between smartphone and wearable device data and affective disorders, Rohani et al. (15) revealed that the association between time spent at home and depressive symptoms was the most consistently significant finding of any smartphone-derived feature in the analysis.

Yet, whilst GPS may provide a valuable source of data to predict symptoms of mental ill-health, there may be certain situations in which GPS data is not available (e.g., due to technological limitations or privacy concerns) or when movement is limited (e.g., due to physical ill-health), requiring us to establish other digital phenotyping data sources to aid the identification of symptoms or risk factors associated with mental disorders.

One plausible source of these additional data points may be consumer wearable devices, such as the Apple Watch (www.apple.com/watch), Fitbit (www.fitbit.com) or Oura Ring (www.ouraring.com). In recent years, the number of connected wearable devices worldwide has proliferated and is expected to exceed 1 billion by 2022 (21). Whilst wearable devices differ in the type and quality of data they collect, common measures include activity (e.g., number of steps and energy expenditure), heart rate and sleep. Individually - and combined - these data points may offer the opportunity for a richer digital phenotyping data set and alternative digital biomarkers in the absence of, or in addition to, valid GPS location data.

The most widely used sensor in wearable devices is the accelerometer, most commonly used to measure an individual's physical activity. There is a large body of research demonstrating the relationship between physical activity and mental health (22–26). In one of the largest studies conducted to-date using wrist-worn devices to measure physical activity in a population-based sample of 2,862 participants, Vallance et al. (27) found a strong association between accelerometer-based activity and decreased rates of depression. In a clinical study of older adults diagnosed with depression, O'Brien et al. (28) found that physical activity was significantly reduced in individuals diagnosed with depression compared to healthy controls.

Decades of research has also demonstrated that sleep alterations are highly prevalent in mental disorders (29–31). A number of sleep markers, including total sleep time, sleep onset latency, sleep efficiency (the ratio of total sleep time to time in bed), and rapid eye movement (REM) have consistently been found to be associated with measures of mental health (32).

Finally, a growing number of wearable devices are now available measuring heart rate variability (HRV). HRV is the variation in time interval between adjacent heart beats (the R-R interval). Typically recorded by an electrocardiogram (ECG) (33), HRV indexes neurocardiac function and is a validated measure of balance in the activity of the autonomic nervous system (ANS) (34, 35). In addition to associations with general cardiovascular health and being a significant predictor of mortality (36), several studies have demonstrated that lower HRV is also associated with increased symptoms of depression (37), anxiety (38), and later stages of bipolar disorder (39). Taken together, consumer wearable devices may therefore provide a valuable source of additional data to help identify moment by moment changes in risk factors associated with mental disorders.

The aim of the current study was to assess to what extent data from smartphone and wearable devices may be used to predict symptoms of depression, anxiety and stress during periods of restricted movement. The study was conducted during the first wave of the coronavirus disease 2019 (COVID-19) pandemic as governments across the world instated widespread restrictions on individual movement and social interaction intended to reduce the incidence of the virus. This provided an opportunity to assess the role of GPS in predicting symptoms of depression and anxiety during periods of limited movement and examine the predictive power of other digital phenotyping data sources.

We sought to answer the following questions:

1. Can location features derived from smartphone GPS data be used to predict symptoms of depression and anxiety? i.e., do previous findings replicate during periods of restricted movement?
2. Can measures of physical activity, sleep and HRV derived from consumer wearable devices predict symptoms of depression and anxiety?
3. Which digital phenotyping variables have the strongest predictive power?
4. What is the difference in predictive power between digital phenotyping data and a daily self-report mood measure in predicting depression and anxiety symptoms?

## Materials and Methods

### Study Design

The current study was a longitudinal observation study with repeated measurements over a 30-day period. Measurements consisted of baseline (day 1), a midpoint (day 16) and endpoint questionnaire (day 31) and daily assessed digital phenotyping variables extracted from smartphone data and wearable data (Oura Ring).

### Participants and Procedure

Participants (*N* = 60) were recruited via posts on online communities and social media sites. Recruitment started on 12 April and was closed on 29 April 2020. Interested individuals were included in the study if they (a) were at least 18 years of age, (b) were able to read and speak English, (c) owned an iPhone with access to the internet, (d) owned an Oura Ring. All participants signed a consent form agreeing with the data collection and analysis. According to the local ethical guidelines in the conduct of research (40), the study was exempt of a formal ethical committee approval since: (1) the study does not deviate from the informed consent; (2) the research does not intervene in the physical integrity of the participants; (3) all our participants are above 15 years old; (4) our study does not expose participants to strong stimuli; (5) there is no intervention nor there is a foreseeable potential for mental harm to the participants that exceed the limits of participants' normal daily life or those around them. As compensation for participating in the study all participants received a personalized mental health and well-being report reviewed by a clinical psychologist (L.B.S.) at the end of the study.

Following completion of the online consent form, participants were emailed an online link to download a custom smartphone application called “Delphi” developed for Apple (iOS) smartphones. The Delphi app was used to gather all study data, including baseline, midpoint and endpoint questionnaire data and data related to participants' daily mood, activity, sleep, HRV, smartphone usage, and context throughout the duration of the study. Participants were required to provide Delphi with permission to access location data at all times, Apple Healthkit and enable notifications from the app. To monitor data collection and compliance during the study period, a secure web-based dashboard was developed displaying the data gathered for all participants, updated at 15-min intervals. Cases of missing data were resolved via email. At the end of the study, participants were requested to uninstall the app.

The present study used methods from a previous study (19) investigating the correlation between GPS variables and depressive symptom severity with a sample size of *N* = 40. However, as dropout rates in longitudinal observation studies using digital phenotyping data are typically high, recruitment was continued for 2 weeks after reaching the required sample size to compensate for dropout.

### Assessment

#### Mental Health Outcomes

Mental health outcomes were assessed at baseline (T0), the midpoint of the study (16 days; T1) and the end of the study (31 days; T2). Symptoms of depression, anxiety and stress were assessed with the Depression Anxiety Stress Scales (DASS-21). The DASS-21 is a 21-item short form of the DASS (41). It measures depressive mood, anxiety, and chronic tension/stress during the past week (e.g., “I was aware of dryness of my mouth”; “I couldn't seem to experience any positive feeling at all.”). All items are rated on a 4-point Likert scale ranging from 0 (“did not apply to me at all”) to 3 (“applied to me very much or most of the time”). The subscores range from 0 to 21, with higher subscores indicating more severe symptoms of depression, anxiety and stress. The DASS21 has demonstrated high internal consistency for the three subscales of depression, anxiety, and stress in previous administrations (42).

To further quantify the baseline severity of participants we used the standard cut-off values for the DASS. The sub-scale “depression” was categorized as normal 0–4, mild 5–6, moderate 7–10, severe 11–13, or extremely severe 14+; the subscale “anxiety” as normal 0–3, mild 4–5, moderate 6–7, severe 8–9, or extremely severe 10+; and the subscale stress as: normal 0–7, mild 8–9, moderate 10–12, severe 13–16, extremely severe 17+ (41, 42).

#### Ecological Momentary Assessment (EMA) of Mood

To assess participant mood, notifications were sent by the Delphi app asking participants to report their mood 3 times per day, randomized within a 30-min window during the morning, afternoon and evening (i.e., ~09:00, 14:30, and 20:00). Mood was assessed through the circumplex model of affect (43), which conceptualizes mood as a two-dimensional construct comprising different levels of valence (positive/negative) and arousal (low/high). We used a single item question, “How are you feeling right now?” and 2 response scales, representing the two dimensions. Levels on both dimensions were tapped on a 9-point scale scored from −4 to 4 (low to high). The default mode was set to zero.

#### Smartphone Sensor Data

Delphi uses the AWARE open source framework (44, 45) to collect raw data from smartphone sensors. Sensors enabled in the current study included Battery, GPS, Screen (on and off), and Timezone. In addition, we used the ESM Scheduler plugin to deliver the EMAs. Supplementary Table 1 provides a detailed list of sensors used in the study, the data collected by each sensor and the sampling frequency.

Data collected by Delphi is first stored locally on the participant's device and then uploaded onto a secure server in the cloud when a WiFi connection is established. To ensure privacy and data protection AWARE obfuscates and encrypts the data using a one-way hashing of logged personal identifiers, such as phone numbers. Increased security is achieved with application permissions, certificates, user authentication, and the use of secure network connections to access and transfer the logged data between the client and the dashboard. For further information on the AWARE framework see Ferreira et al. (44) and Nishiyama et al. (45).

#### Activity, Sleep, and HRV Data

We used the Oura Ring to measure participants' activity, sleep and HRV. Activity measures included number of steps (“step count,” measured via the device's 3D accelerometer) and metabolic equivalent for task (MET). MET is a standardized measurement of the amount of energy used by the body during physical activity, as compared to resting metabolism (46). One MET is defined as the energy the body uses at rest. In the current study we used an average score to determine the energy expenditure during a 24-h period.

To measure sleep, the Oura Ring uses a combination of accelerometer data, heart rate, HRV and pulse wave variability amplitude in combination with machine learning models to calculate deep (N3), light (N1+N2) and rapid-eye-movement (REM) sleep in addition to sleep/wake. The Oura Ring has been shown to have high agreement with polysomnography (PSG; the gold-standard for measuring sleep) in the whole night estimation of total sleep time (TST), sleep onset latency (SOL) and wake after sleep onset (WASO) (47). For the current study we measured participants' TST, SOL, WASO and time in bed (TIB).

We also used the Oura Ring to measure participants' average night-time heart rate variability (HRV). The Oura Ring calculates HRV using the root mean square of successive differences between normal heartbeats (RMSSD). Although the R-peak detection typical of ECG is not directly available via the Oura Ring, the device has been shown to have high agreement (*r*^2^ = 0.98) with ECG (the gold standard for measuring HRV) (48).

### Data Processing

#### Data Preprocessing

We converted the UNIX timestamps of each sensor data into a human-readable local date and time format using each participant's timezone data. We then aggregated the data at the “day” level. To ensure location accuracy, we removed all duplicate entries in the database as well as GPS coordinates with accuracy > 80th percentile of all participants' GPS accuracies and GPS coordinates with latitude 0.0 and longitude 0.0 that arose due to sensing errors.

Preprocessing and extraction of the location features were computed according to Saeb et al. (19). Prior to feature extraction we established whether each GPS location data sample represented a stationary state (e.g., at home) or transition state (e.g., walking outside). This was determined by calculating the movement speed at each location sample using its time derivative. A movement speed > 1 km/h was defined as a transition state. We then applied a K-means clustering algorithm (49) to the stationary state data samples to identify the locations where participants spent the majority of their time.

#### Location Feature Extraction

We extracted five location features from the GPS data: Total Distance, Location Variance, Entropy, Normalized Entropy and Time at Home.

*Total Distance* was defined as the total number of kilometers traveled by the participant during the specified time period. It was calculated as the sum score of the distances between the location samples.

*Location Variance* was defined as the variability in participants' GPS locations. It was calculated from the logarithm of the sum of the variance in latitude and longitudinal coordinates of the stationary states.

*Location Entropy* was defined as the variability of the time participant spent at the location clusters. It was computed as: *[Entropy* = –∑_*i*_*p*_*i*_log *p*_*i*_] where each *i* = 1, 2, …, *N* represented a location cluster, *N* represented the total number of location clusters, and *p*_*i*_ represented the percentage of time spent at the location cluster. Higher entropy reflected the fact that the participant spent similar amounts of time at different clusters (e.g., 50% of time at home and 50% of time at work), lower entropy reflected that participants spent significantly more time at certain clusters than others (e.g., 70% of time at work, 30% of time at home).

*Normalized Location Entropy* was computed to provide a measure of entropy that is invariant to the number of clusters a participant spent time at. It was calculated by dividing the entropy by its maximum value, which is the logarithm of the total number of clusters. The resulting value ranges from 0 to 1, where 0 represents that all location data points belong to the same cluster and 1 indicates that they are uniformly distributed across all clusters.

*Time at Home* was defined as the proportion of time a participant spent at home relative to other location clusters. To calculate it we first defined the home cluster as the cluster with the most GPS coordinates between the hours of 00:00 and 06:00. We then computed the percentage of time by dividing the total time spent in the home cluster by 24 h.

#### Phone Usage Feature Extraction

We extracted two features related to phone usage. First, *Phone Usage Frequency* was defined as the number of times a participant interacted with their phone during the specified time period. Interactions were calculated based on a screen unlocking event. Second, *Phone Usage Duration* was defined as the total number of minutes a participant interacted with their phone during the specified time period. The usage session duration was calculated as the time from when phone is unlocked until it was locked.

### Statistical Analysis

Before beginning the analyses, study dates were converted into the study day (1–30) specific to each participant. The extracted smartphone features, wearable data, EMA data (independent variables) and scores on the DASS-21 subscales (depression, anxiety, stress; dependent variables) were then synchronized according to the study day.

#### Correlation Analysis

To ensure that all variables in the analyses reflected the same time period, the daily smartphone and wearable feature data was pooled for the first 2 weeks and second 2 weeks of the study to align with the timing of the DASS-21 measurements. For example, we calculated the average Total GPS Distance during days 1–15 and correlated this with the DASS-21 scores at T1 (day 16) and calculated the average Total GPS Distance during days 16–30 and correlated this with the DASS-21 scores at T2 (day 31). According to this, all features were pooled and correlations with the respective DASS-21 were investigated (average feature data from day 1–15 with T1 DASS-21 and average feature data from day 16–30 with T2 DASS-21). Correlations were calculated using the Spearman's correlation coefficient, since the data was not normally distributed. *P*-values were adjusted for multiple testing based on the Bonferroni Holm method (50) with a false discovery rate of 0.05. To avoid biases introduced by missing values we used full information maximum likelihood as the estimator (51, 52). However, *p*-value adjustment methods are sensitive and since the present study is of an exploratory nature, adjustment was only performed cluster-wise (e.g., separately for GPS features and wearable features) to avoid an overcorrection by *p*-value adjustment leading to a false-rejection of findings.

#### Predicting Mental Health Symptom Severity From Smartphone and Wearable Data

To account for the hierarchical structure of the data we used a multilevel model (MLM) to predict the influence of smartphone and wearable data on mental health scores (53–55). MLMs take into account that data is nested within persons, i.e., the observations are not independent (56) and reduce the likelihood of Type I errors (57). In the current study, the repeated measures (level 1) are nested within the person (level 2). Intra-class correlations (ICC) underlined the necessity of a MLM (all ICC > 0.05).

To investigate whether mental health symptom severity can be predicted from smartphone and wearable device data, we pooled the data in the same manner as the correlation analyses and applied the MLM with random intercepts and random slopes to four sets of independent variables: GPS features (total distance, location variance, entropy, normalized entropy, and time at home); smartphone usage features (usage duration and usage frequency); wearable device data (step count, MET, TST, SOL, WASO, TIB, and HRV); and EMA mood data (valence and arousal). Variables were z-standardized.

The intercept represents the average depression, anxiety, stress scores across the study and the slope represents the association between mental health scores and the smartphone and wearable data. Two-sided *p* < 0.05 were considered statistically significant.

In a first step, regression models were built separately for each predictor to investigate its predictive power on depression, anxiety and stress scores. In a second step we explored whether the combination of multiple predictors could outperform single predictor models. Only predictors showing predictive power in single predictor models were included in the combined model. All models were fitted using maximum likelihood. Combined models with different predictors were compared to each other to investigate whether more complex models with more predictors were superior. For the comparison of the models, likelihood ratio tests were used (51, 58, 59).

#### Missing Data Handling

Missingness only occurred in the DASS-21 assessment (10%) and the sensing variables (9.1%) and was assumed to be missing at random (MAR), meaning missingness depended on observed data (51, 52). To avoid bias introduced by missingness we used multiple imputations to handle missing values in the correlation and regression analysis. The imputation model took the nested structure of the data into account and followed guidelines for multilevel multiple imputations (58). To make the MAR assumption hold, variables related to non-response and explaining variance in observed variables were included in the imputation model. Predictive mean matching for multilevel was used as imputation method. The number of imputed data sets was set to 20 and the number of iterations to 15. Convergence was visually assessed and confirmed (58). Regression analysis was performed on each imputed data set and results were pooled using the Rubin's rule (60).

### Software

The pre-processing and feature extraction were performed using Python (version 3.7.6), R (version 4.0.2) (61), snakemake (version 5.20) (62) and following workflow examples from RAPIDS (63). All analyses were conducted in R. Correlations were calculated using the “psych” package (52). MLM was carried out using the packages lme4 (64) and lmerTest (65). For multiple imputations the “MICE” package was used (58).

## Results

### Participant Characteristics and Adherence

Of the 60 participants at intake, 1 participant (1.7%) dropped out of the study due to concerns over privacy, 2 participants (3.4%) dropped out due to burden of self-report and 2 (3.4%) participants dropped out for unknown reasons. Of the remaining 55 participants, 47 (85.5%) completed the midpoint questionnaire and 54 (98.2%) completed the endpoint questionnaire.

Of the 55 participants, 30 (54.5%) were female and 25 (45.5%) were male. Their ages ranged from 24 to 68 with a mean of 42.8 (SD 11.6). There were 2% with no secondary education, 17% with high school as the highest education, and 80% with bachelor's degree or a higher degree. The mean depression severity was M = 3.78, SD = 3.48 (normal: 67.3%, mild-moderate: 25.4%, severe: 7.3%), the mean anxiety severity was M = 2.73, SD = 2.68 (normal: 70.9%, mild-moderate: 23.7%, severe: 5.5%), and the mean stress level was M = 6.00, SD = 3.82 (normal: 65.5%, mild-moderate: 29.1%, severe: 4.4%). Table 1 provides a detailed summary of all participants included in the final analysis.

**Table 1:** Participant characteristics.

**Variable**	**%/M (SD)**	***n***
Age	42.8 (11.6)	55
**Gender**		
Female	54.5	30
Male	45.5	25
**Ethnicity**		
Asian/Pacific Islander	1.8	1
Hispanic or Latino	3.6	2
White	92.7	51
Other	1.8	1
**Education**		
Less than a high school diploma	1.9	1
High school degree or equivalent	16.7	9
Bachelor's degree (e.g., BA, BS)	44.4	24
Master's degree (e.g., MA, MS)	33.3	18
Doctorate (e.g., PhD, EdD)	1.9	1
Prefer not to say	1.9	1
**Employment**		
Employed full-time (35+ h a week)	49.1	27
Employed part-time (<35 h a week)	7.3	4
Unemployed (currently looking for work)	1.8	1
Unemployed (not currently looking for work)	3.6	2
Student	5.5	3
Retired	1.8	1
Self-employed	21.8	12
Unable to work	7.3	4
Prefer not to say	1.8	1
**Marital status**		
Single (never married)	25.5	14
Married	49.1	27
In a domestic partnership	20.0	11
Divorced	5.5	3
**Mental health status at baseline**		
Depression	3.78 (3.48)	–
Normal	67.3%	37
Mild	12.7%	7
Moderate	12.7%	7
Severe	5.5%	3
Extremely severe	1.8%	1
Anxiety	2.73 (2.68)	–
Normal	70.9%	39
Mild	16.4%	9
Moderate	7.3%	4
Severe	1.8%	1
Extremely severe	3.6%	2
Stress	6.00 (3.82)	–
Normal	65.5%	36
Mild	18.2%	10
Moderate	10.9%	6
Severe	3.6%	2
Extremely severe	1.8%	1

*Values are based on observed data*.

The majority of participants (66%) were from Finland. During the time of the study, the restrictions in Finland were such that the government strongly recommended that individuals maintain social distancing, companies adopt remote work wherever possible and the majority of public and private facilities (e.g., libraries, museums, bars and sports facilities) were temporarily closed. A multi-level model predicting the daily distance traveled (using categorized country Finland vs. other as predictor) revealed no significant distance between the average daily distance traveled between participants from Finland and those from other countries (β = −922.9 [in meters], *p* = 0.943).

### Correlations Between Smartphone and Wearable Data and Mental Health Symptoms

Table 2 presents Pearson's correlation matrixes of the four sets of independent variables (GPS, smartphone-usage, wearable, and EMA features) and mental health symptom severity.

**Table 2:** Correlations between smartphone and wearable data and mental health scores.

	**Depression**		**Anxiety**		**Stress**	
	***r***	***p***	***r***	***p***	***r***	***p***
**GPS features**						
Location variance	−0.31	0.035^*^	−0.26	0.110	−0.28	0.077
Total distance	−0.26	0.110	−0.28	0.077	−0.17	0.572
Location entropy	−0.30	0.035^*^	−0.17	0.572	−0.22	0.251
Normalized location entropy	−0.26	0.100	−0.13	0.788	−0.20	0.298
Homestay	0.12	0.788	0.13	0.788	0.07	0.788
**Smartphone usage features**						
Usage time	0.09	> 0.99	0.05	> 0.99	0.07	> 0.99
Usage frequency	0.15	0.716	0.24	0.079	0.12	0.971
**Wearable device data**						
Steps	−0.23	0.516	−0.16	> 0.99	−0.19	> 0.99
Metabolic equivalent for task	−0.21	0.99	−0.07	> 0.99	−0.06	> 0.99
Total sleep time	0.15	> 0.99	0.04	> 0.99	0.12	> 0.99
Sleep onset latency	0.08	> 0.99	0.06	> 0.99	0.04	> 0.99
Wake after sleep onset	0.20	> 0.99	0.14	> 0.99	0.16	> 0.99
Time in bed	0.15	> 0.99	0.03	> 0.99	0.11	> 0.99
Heart rate variability	0.08	> 0.99	0.12	> 0.99	0.09	> 0.99
**Mood**						
Arousal	−0.30	0.003^*^	−0.35	0.001^*^	−0.36	0.001^*^
Valence	−0.48	<0.001^*^	−0.44	<0.001^*^	−0.48	<0.001^*^

*All p-values are adjusted cluster-wise for multiple testing*;

* *indicates significance*.

For all three mental health outcomes, significant small-to-medium correlations with the obtained EMA data – valance and arousal - were found (see Table 2). In contrast, none of the wearable or smartphone usage features were associated with mental health symptom severity in the correlation analysis (p>0.05). Also, for GPS features only the location variance and the entropy showed a significant correlation with depression. All other GPS features as well as variance and entropy for anxiety and stress were non-significant (see Table 2 for more details).

### Predicting Symptom Severity From Smartphone and Wearable Data

Analyses of the GPS-derived location features showed that location variance had a negative association with subsequent depressive symptom severity (beta = −0.21, SE = 0.10, *t*(81) = −2.13, *p* = 0.037), but no significant relationship with symptoms of anxiety or stress. No significant association between the other GPS-derived features (total distance, location entropy, normalized location entropy and time at home) and symptoms of depression, anxiety or stress were found.

With regards to smartphone usage features, we found no significant relationship between smartphone usage duration or smartphone usage frequency and symptoms of depression, anxiety and stress.

The analyses of wearable device data showed no significant association between any of the physical activity measures (MET and steps) and depression, anxiety, and stress. From the sleep measures, we found a significant relationship between total sleep time and depression [beta = 0.24, SE = 0.11, *t*(73) = 2.33, *p* = 0.023], time in bed and depression [beta = 0.26, SE = 0.11, *t*(59) = 2.39, *p* = 0.020] and WASO and anxiety [beta = 0.23, SE = 0.11, *t*(90) = 2.13, *p* = 0.035]. Additionally, we found a significant association been HRV and anxiety [beta = 0.26, SE = 0.12, *t*(71) = 2.15, *p* = 0.035]. None of the sleep measures were significantly related to stress.

The EMA data (valence and arousal) showed that valence was significantly related to depression [beta = −0.39, SE = 0.11, *t*(55) = −3.43, *p* = 0.001], anxiety [beta = −0.30, SE = 0.12, *t*(57) = −2.54, *p* = 0.014], and stress [beta = −0.39, SE = 0.11, *t*(74) = −3.64, *p* < 0.001]. There was no significant relationship between arousal and depression, anxiety or stress. Supplementary Tables 2–4 provide the full set of results from single predictors.

### Combined Predictions

Depression could be predicted by EMA and smartphone and wearable data. EMA performed better than smartphone and wearable data models, but the combination yielded the best fit (see Table 3). For anxiety and stress, EMA-only data models were the strongest predictors (see Tables 4, 5).

**Table 3:** A comparison of MLM model performance on the prediction of depression.

**Model**	**Fixed effects**					Goodness of fit and Comparison^*^		
	**Estimate**	**SE**	***t*-value**	**Df**	***p*-value**	**F**	**df1, df2**	***P*-value**
**Baseline model**								
Intercept	0	0.13	0	105	> 0.999			
**EMA model**								
Intercept	0	0.11	0.00	104	> 0.999	10.52	1, 172	0.001^a^
Valence	−0.39	0.11	−3.49	55	0.001			
**GPS model**								
Intercept	0	0.12	0	104	> 0.999	4.574751	1, 568	0.033^a^
Variance	−0.21	0.10	−2.15	81	0.035			
**Extended digital phenotyping model: GPS and wearable data**								
Intercept	0	0.11	0.00	103	> 0.999	5.23	1, 136	0.024^b^
Variance	−0.21	0.10	−2.17	78	0.033			
Time in bed	0.25	0.10	2.43	58	0.018			
**Combined model: EMA and digital phenotyping model**								
Intercept	0	0.10	0.00	102	> 0.999	11.47	1, 176	0.001^c^
Variance	−0.21	0.09	−2.32	72	0.023	5.42	2, 560	0.005^d^
Time in bed	0.24	0.09	2.54	59	0.014			
Arousal	−0.38	0.10	−3.61	52	0.001			

* *For more details on the likelihood ratio test see chapter 5.3.1 in Van Buuren (66)*.

a *Comparison against baseline*,

b *Comparison against GPS*,

c *Comparison against extended sensing model*,

d *Comparison against EMA model*.

**Table 4:** A comparison of MLM model performance on the prediction of anxiety.

**Model**	**Fixed effects**					Goodness of fit and comparison^*^		
	**Estimate**	**SE**	**t**	**df**	***p*-value**	**F**	**df1, df2**	***P*-value**
**Baseline model**								
Intercept	0	0.12	0.00	105	> 0.999			
**EMA model**								
Intercept	0	0.11	0.00	104	> 0.999	5.63	1, 191	0.019^a^
Valance	−0.30	0.12	−2.60	57	0.012			
**GPS model**								
No predictors identified								
**Wearable data model**								
Intercept	0	0.12	0.00		> 0.999	4.45	1, 997	0.035^b^
WASO	0.23	0.11	2.16		0.034			
**Combined model: EMA and digital phenotyping model**								
Intercept	0	0.11	0.00	103	> 0.999	5.03	1, 240	0.026^c^
Valence	−0.27	0.11	−2.41	59	0.001	3.67	1, 2,655	0.055^d^
WASO	0.19	0.10	1.90	90				

* *For more details on the likelihood ratio test see chapter 5.3.1 in Van Buuren (66)*.

a *Comparison against baseline*,

b *Comparison against GPS*,

c *Comparison against extended sensing model*,

d *Comparison against EMA model*.

**Table 5:** A comparison of MLM model performance on the prediction of stress.

**Model**	**Fixed effects**					Goodness of fit and comparison^*^		
	**Estimate**	**SE**	**t**	**df**	***p*-value**	***F***	**df1, df2**	***P*-value**
**Baseline model**								
Intercept	0	0.12	0.00	105	> 0.999	282.3	290.4	–
**EMA model**								
Intercept	0	0.10	0.00	104	> 0.999	11.09	1, 434	0.001^a^
Arousal	−0.39	0.11	−3.71	74	<0.001			
**GPS model**								
No predictors identified								
**Wearable data model**								
No predictors identified								
**Combined model: EMA and digital phenotyping model**								
Not applicable								

* *For more details on the likelihood ratio test see chapter 5.3.1 in Van Buuren (66)*.

a *Comparison against baseline*.

## Discussion

The current study assessed whether data from smartphone and wearable devices could predict symptoms of depression and anxiety during periods of limited movement. We found that GPS (location variance on depression) and wearable device data (total sleep time and time in bed on depression; wake after sleep onset and HRV on anxiety), were able to predict mental health. Furthermore, a combined model of GPS and wearable data significantly increased the ability to predict symptoms of depression and anxiety compared to GPS data alone.

The finding that greater diversity in visited locations predicted lower depression severity supports previous research demonstrating that participants who move about more through geographic space are less depressed (19, 67). Furthermore, it indicates that, despite limited movement and social interaction, GPS may still provide a valuable source of data for the identification of individuals at risk of developing mental disorders. However, contrary to previous findings (19, 67–69), we did not find a significant relationship between the other smartphone features (total distance, location entropy, normalized location entropy and time spent at home) and mental health measures. This may be explained by weaker associations found in the current study compared to previous studies or by abnormal movement patterns during COVID-19. As such, it highlights the importance of further research on the role of context and other moderating variables that may influence the relationship between different GPS-derived features and mental health.

From the physiological data derived from the wearable device, we found total sleep time and time in bed to be significant predictors of depressive symptom severity. One explanation for this may be the lack of motivation and fatigue exhibited by individuals suffering from depression. The additional finding that longer periods of wakefulness after falling asleep significantly predicted symptoms of anxiety may be explained by the hyper-vigilance or hyperarousal characteristic of anxiety disorders causing individuals to wake up more frequently during their sleep (70). Similar findings to these have been reported in previous studies using polysomnography in a laboratory setting (32), however this is the first study to use validated consumer wearable devices to provide sleep data in real-life settings. Given the transdiagnostic nature of sleep disturbances (30) and that insomnia has been identified as a precursor to the development of full clinical syndromes (71), sleep data from consumer wearables may thus provide valuable tools to identify early warning signs of mental disorders, thereby facilitating time-sensitive preventative measures (72).

Finally, the superior performance of the combined GPS and wearable data model compared to the GPS-only model in predicting symptoms of depression and anxiety demonstrates the value of wearable data during times of restrictive movement such as COVID-19. Furthermore, our finding that adding smartphone and wearable data to EMA data had the highest predictive power of all models in the analysis suggests that a combination of passive sensing and active assessment may provide the greatest predictive power in identifying people suffering from symptoms of depression and anxiety.

A number of limitations of the study should be taken into account. First, it is important to highlight that this is a longitudinal observational study, thus the current findings do not necessarily represent a causal relationship between the behavior measured by smartphone and wearable devices and symptoms of depression and anxiety, nor can they explain the direction between them. For example, an increase in total time in bed may be a cause or effect of the increase in depressive symptom severity or it may be explained by another third variable (73). Furthermore, as pooling data prevented us from exploring high-frequency processes (e.g., the relationship between movement and mood), we were unable to establish temporal precedence (74). Second, the small sample size meant that the study was likely underpowered to find statistically significant results for a number of predictors exhibiting small effect sizes. Forthcoming, studies should therefore assess whether the current findings are replicated in a larger sample size. Third, there were some sample biases. The sample was heavily skewed toward white, employed individuals who were more educated than the general population. Related to this, the study was open only to participants with an Apple (iOS) smartphone. Research has shown that sociodemographic status and smartphone usage behavior may differ between iOS and Android users (75, 76). Future studies should therefore assess the relationship between digital phenotyping data and mental health across both platforms and in populations with more diverse backgrounds. Forth, participants were a non-clinical sample recruited from the general population. This was intentional as the focus of the current study was related to the detection of depression across a continuous spectrum and in a naturalistic setting. Although participants scored highly on the depression subscale in particular (over 25% of participants had at least mild-to-moderate depression severity), clinical diagnosis was not an inclusion criterion. Future research would therefore benefit from examining the relationship between the current smartphone and wearable device data and mental health in a clinical population. Finally, individual responses to restrictions on movement during the COVID-19 pandemic are likely to have varied considerably. As the study commenced after the onset of the pandemic, we were unable to provide any data comparing participants' movement before and during the study to confirm that their movement was indeed more limited during the study period.

Notwithstanding these limitations, the current study provides promising indications of digital phenotyping data derived from consumer wearable devices for the identification of individuals suffering from or at risk of developing a mental health condition. Future research would benefit from assessing how data from additional sensors [e.g., speech and voice (77), keyboard interactions (78), bio-sensing (79), and smartphone app usage (80)], combined with machine learning models may be used to further improve predictive accuracy (81–85). Given the issues quantifying explained variance in multilevel models (86, 87), future research would also benefit from understanding the amount of variance explained in symptomatology by smartphone and wearable sensing data. Studies with larger samples sizes, conducted over longer periods of time are also needed, both to ensure adequate power as well as to assess how digital phenotypes may be used to predict changes in symptomatology over time (88, 89). Such research may also provide valuable insights into the causal mechanisms underlying mental disorders (e.g., behavioral activity, loneliness) and thereby enable the development of early mental health warning systems and more effective, timely interventions targeted to the individual based on personalized models of psychopathology (90, 91).

## Data Availability Statement

The datasets presented in this article are not readily available because the data we report are from unique patients and therefore identifiable when the full set of data is provided. However, the code used to generate the data may be provided upon reasonable request to the corresponding author.

## Ethics Statement

Ethical review and approval was not required for the study on human participants in accordance with the local legislation and institutional requirements. The patients/participants provided their written informed consent to participate in this study.

## Author Contributions

IM, LP-R, DM, DF, KA, LS, and YT contributed to the study conceptualization and design. IM, KA, DF, and YT participated in data collection. YT, LS, KA, and IM contributed to the creation of participant reports. KA, DF and IM contributed to feature extraction. YT, IM, KA, DF, DM, LS, and LP-R contributed to the methods and analysis. IM, YT, and KA prepared the original draft. LP-R, DM, HB, DF, and LS critically reviewed and edited the draft. All authors read and approved the final manuscript and account for all aspects of the work.

## Additional Information

Correspondence and requests for materials should be addressed to IM.

## Code Availability

The code used to process and analyze the findings of this study may be made available to an investigator upon reasonable request.

## Conflict of Interest

The authors declare that the research was conducted in the absence of any commercial or financial relationships that could be construed as a potential conflict of interest.
