# Supplementary material for: Predicting Symptoms of Depression and Anxiety Using Smartphone and Wearable Data
Source: Front Psychiatry. 2021 Jan 28;12:625247. doi: 10.3389/fpsyt.2021.625247 (PMC7876288; doi:10.3389/fpsyt.2021.625247)
Supplement: Supplementary file 1 [file Table_1.DOCX]

**Supplementary Material**

**Supplementary Table 1.** Smartphone sensor data collected via Delphi App

**Supplementary Table 2.** Single predictor summary for Depression

**Supplementary Table 3.** Single predictor summary for Anxiety

**Supplementary Table 4.** Single predictor summary for Stress

**Supplementary Table 1. *Smartphone sensor data collected via Delphi App***

| **Sensor** | **Data collected** | **Sampling Frequency** |
| --- | --- | --- |
| Battery | Initiation of phone charging, unplugging of device, battery level | When battery information has changed and/or the device starts to charge, is unplugged or has finished charging |
| ESM |  | 3 times per day (see section entitled EMA for more details) |
| Locations | Participant’s current location based on GPS latitude and longitude coordinates | 5 minutes |
| Screen / Device Usage plugin | Phone usage and non-usage sessions measured using the screen sensor. A usage session is defined as the time from when phone is unlocked until it is locked. Non-usage is defined as the time from when phone is locked until it is unlocked. | As detected by sensors |
| Timezone | User’s current timezone based on GPS at the time of measurement | 60 minutes |

**Supplementary Table 2. *Single predictor summary for Depression***

| **Predictor** | **Estimate** | **SE** | **t** | **df** | **p-value** | **AIC** | **BIC** |
| --- | --- | --- | --- | --- | --- | --- | --- |
| **GPS features** |  |  |  |  |  |  |  |
| Location Variance | -0.21 | 0.10 | -2.13 | 81 | .037 | 272.91 | 283.71 |
| Total Distance | -0.09 | 0.11 | -0.80 | 61 | .425 | 277.14 | 287.94 |
| Location Entropy | -0.09 | 0.09 | -1.01 | 99 | .313 | 277.36 | 288.16 |
| Normalized Location  Entropy | -0.05 | 0.08 | -0.59 | 101 | .559 | 287.37 | 289.17 |
| Homestay | 0.08 | 0.08 | 1.05 | 91 | .295 | 277.47 | 288.27 |
| **Smartphone usage features** |  |  |  |  |  |  |  |
| Usage frequency | 0.11 | 0.10 | 1.08 | 91 | .285 | 276.87 | 287.67 |
| Usage time | 0.05 | 0.12 | 0.41 | 98 | .680 | 277.70 | 288.50 |
| **Wearable device data** |  |  |  |  |  |  |  |
| Steps | -0.22 | 0.12 | -1.80 | 51 | .077 | 273.18 | 283.98 |
| Metabolic equivalent  for task | -0.20 | 0.12 | -1.58 | 50 | .121 | 274.15 | 284.95 |
| Total sleep time | 0.24 | 0.11 | 2.33 | 73 | .023 | 271.65 | 282.45 |
| Sleep onset latency | 0.08 | 0.09 | 0.84 | 78 | .401 | 277.53 | 288.33 |
| Wake after sleep  onset | 0.12 | 0.12 | 0.95 | 49 | .346 | 276.52 | 287.32 |
| Time in bed | 0.26 | 0.11 | 2.39 | 59 | .020 | 270.67 | 281.48 |
| Heart rate variability | 0.18 | 0.15 | 1.23 | 35 | .226 | 274.61 | 285.41 |
| **EMA** |  |  |  |  |  |  |  |
| Valence | -0.39 | 0.11 | -3.43 | 55 | .001 | 263.73 | 274.53 |
| Arousal | -0.18 | 0.11 | -1.60 | 61 | .114 | 274.98 | 285.78 |

**Supplementary Table 3. *Single predictor summary for Anxiety***

| **Predictor** | **Estimate** | **SE** | **t** | **df** | **p-value** | **AIC** | **BIC** |
| --- | --- | --- | --- | --- | --- | --- | --- |
| **GPS features** |  |  |  |  |  |  |  |
| Location Variance | -0.16 | 0.11 | -1.44 | 63 | .155 | 285.4 | 296.2 |
| Total Distance | -0.07 | 0.11 | -0.65 | 68 | .520 | 287.66 | 298.47 |
| Location Entropy | -0.07 | 0.10 | -0.72 | 82 | .474 | 287.84 | 298.64 |
| Normalized Location  Entropy | -0.00 | 0.09 | -0.03 | 89 | .978 | 288.75 | 299.55 |
| Homestay | 0.10 | 0.10 | 1.20 | 97 | .235 | 287.31 | 298.11 |
| **Smartphone usage features** |  |  |  |  |  |  |  |
| Usage frequency | 0.14 | 0.11 | 1.26 | 75 | .210 | 286.29 | 297.10 |
| Usage time | 0.06 | 0.12 | 0.47 | 98 | .639 | 287.87 | 298.67 |
| **Wearable device data** |  |  |  |  |  |  |  |
| Steps | -0.9 | 0.12 | -0.75 | 65 | .457 | 287.37 | 298.17 |
| Metabolic equivalent  for task | -0.09 | 0.11 | -0.84 | 97 | .402 | 287.52 | 298.32 |
| Total sleep time | 0.13 | 0.11 | 1.13 | 75 | .261 | 286.79 | 297.59 |
| Sleep onset latency | 0.01 | 0.09 | 0.12 | 94 | .907 | 288.56 | 299.37 |
| Wake after sleep  onset | 0.23 | 0.11 | 2.13 | 90 | .035 | 283.35 | 294.15 |
| Time in bed | 0.19 | 0.11 | 1.73 | 73 | .088 | 284.94 | 295.74 |
| Heart rate variability | 0.26 | 0.12 | 2.15 | 71 | .035 | 282.43 | 293.23 |
| **EMA** |  |  |  |  |  |  |  |
| Valence | 0.30 | 0.12 | -2.54 | 57 | .014 | 280.73 | 291.53 |
| Arousal | -0.23 | 0.12 | -1.90 | 51 | .063 | 283.36 | 294.17 |

**Supplementary Table 4. *Single predictor summary for Stress***

| **Predictor** | **Estimate** | **SE** | **t** | **df** | **p-value** | **AIC** | **BIC** |
| --- | --- | --- | --- | --- | --- | --- | --- |
| **GPS features** |  |  |  |  |  |  |  |
| Location Variance | -0.14 | 0.10 | -1.34 | 90 | .184 | 287.44 | 298.25 |
| Total Distance | -0.09 | 0.14 | -0.65 | 34 | .522 | 287.90 | 298.70 |
| Location Entropy | -0.01 | 0.10 | -0.09 | 83 | .925 | 289.45 | 300.25 |
| Normalized Location  Entropy | 0.08 | 0.09 | 0.91 | 81 | .364 | 288.76 | 299.56 |
| Homestay | 0.05 | 0.08 | 0.59 | 99 | .554 | 289.45 | 300.25 |
| **Smartphone usage features** | 0.10 |  |  |  | .347 | 288.41 | 299.21 |
| Usage frequency | 0.10 | 0.11 | 0.94 | 101 | .399 | 288.34 | 299.14 |
| Usage time | 0.10 | 0.12 | 0.85 | 99 | 0.400 | 288.34 | 299.14 |
| **Wearable device data** |  |  |  |  |  |  |  |
| Steps | -0.16 | 0.12 | -1.40 | 65 | .167 | 286.60 | 297.40 |
| Metabolic equivalent  for task | -0.11 | 0.12 | -0.97 | 74 | .335 | 287.95 | 298.75 |
| Total sleep time | 0.12 | 0.11 | 1.09 | 71 | .281 | 287.74 | 298.54 |
| Sleep onset latency | 0.01 | 0.10 | 0.12 | 68 | .907 | 289.35 | 300.15 |
| Wake after sleep  onset | 0.11 | 0.11 | 1.02 | 86 | .311 | 288.06 | 298.86 |
| Time in bed | 0.14 | 0.11 | 1.29 | 76 | .201 | 287.32 | 298.12 |
| Heart rate variability | 0.14 | 0.13 | 1.12 | 68 | .269 | 287.42 | 298.22 |
| **EMA** |  |  |  |  |  |  |  |
| Valence | -0.39 | 0.11 | -3.64 | 74 | <.001 | 276.26 | 287.06 |
| Arousal | -0.18 | 0.12 | -1.54 | 68 | .127 | 286.74 | 297.54 |
